# MRI of Total Hip Arthroplasty: Successful Ripple Artifact Reduction Using Wavelet Domain Filtering

**DOI:** 10.1002/jmri.70448

**Published:** 2026-07-11

**Authors:** Jeanette C. Deck, Sophia S. Goller, Georg W. Kajdi, Adrian A. Marth, Patrick Zingg, Constantin von Deuster, Reto Sutter

**Affiliations:** ^1^ Department of Radiology Balgrist University Hospital Zurich Switzerland; ^2^ Medical Faculty University of Zurich (UZH) Zurich Switzerland; ^3^ Swiss Innovation Hub (SIH), Siemens Healthineers International AG Zurich Switzerland; ^4^ Department of Orthopedic Surgery Balgrist University Hospital Zurich Switzerland; ^5^ Swiss Center for Musculoskeletal Imaging (SCMI), Balgrist Campus AG Zurich Switzerland

**Keywords:** artifact reduction, magnetic resonance imaging, metal artifact, multispectral imaging, total hip arthroplasty

## Abstract

**Background:**

Slice encoding for metal artifact correction (SEMAC) is used in MRI for total hip arthroplasties for metal artifact reduction. However, ripple artifacts often arise near the implant, impairing diagnosis in affected regions.

**Hypothesis:**

Applying a wavelet domain filter (WD‐Filter) might reduce ripple artifacts and improve image quality.

**Study Type:**

Retrospective.

**Subjects:**

100 patients with primary total hip arthroplasty (female: 51, mean age: (64 ± 13) years; male: 49, mean age: (65 ± 13) years) who underwent clinically indicated MRI using compressed sensing SEMAC.

**Field Strength/Sequences:**

1.5 T; compressed sensing‐accelerated turbo spin‐echo (TSE)‐based SEMAC sequence.

**Assessment:**

Three readers rated 250 ripple artifacts before and after applying the WD‐filter. 4‐point Likert scales were used for the intensity of ripple artifacts (Likert‐RA, “none”: 1, “mild”: 2, “moderate”: 3, “severe”: 4) and the image quality (Likert‐IQ, “poor”: 1, “moderate”: 2, “good”: 3, “excellent”: 4). The relative reduction of the signal intensity standard deviation ($\Delta SD_{\mathrm{ROI}}$) was measured in a region of interest (ROI) containing the ripple artifact.

**Statistical Tests:**

Inter‐reader agreement was assessed with Fleiss' kappa, with 95% confidence intervals. Differences between pre‐ and post‐filter ratings (Likert‐RA and Likert‐IQ) were evaluated using the Wilcoxon signed‐rank test, while $\Delta SD_{\mathrm{ROI}}$ was compared using a two‐sided paired *t*‐test. A *p* value of less than 0.05 indicated statistical significance.

**Results:**

Prior to WD‐Filter application, $\left(122\pm 2\right)$ ripple artifacts were rated as “severe”, $\left(95\pm 3\right)$ as “moderate”, and $\left(32\pm 1\right)$ as “mild”. After filtering, $\left(168\pm 4\right)$ artifacts were rated as “none”, and $\left(82\pm 4\right)$ as “mild”. Upon filter application, Likert‐RA and Likert‐IQ significantly improved from $\left(3.4\pm 0.7\right)$ to $\left(1.3\pm 0.5\right)$ and $\left(1.4\pm 0.5\right)$ to $\left(3.4\pm 0.6\right)$, respectively, with a large effect size (*r* > 0.89) and “almost perfect” inter‐rater agreement (kappa range: 0.86–0.96). The filtered images showed a significantly reduced $\Delta {\mathrm{SD}}_{\mathrm{ROI}}$ compared to the unfiltered images (relative change: $\left(19\%\pm 10\%\right)$).

**Data Conclusion:**

The WD‐Filter effectively reduced ripple artifacts in MRI of total hip arthroplasty and improved image quality.

**Evidence Level:**

3.

**Technical Efficacy:**

Stage 3.

## Plain Language Summary

MRI of patients with metal hip implants often produces wave‐like image distortions called ripple artifacts, which can interfere with the visualization of surrounding tissues and impair diagnosis. The proposed wavelet domain filter (WD‐Filter) removes these artifacts from the images after the scan, without requiring additional imaging time. In a study of 100 patients with hip replacements, three radiologists independently confirmed that the filter significantly reduced ripple artifacts and improved image quality. No cases showed worsened image quality after filtering. This post‐processing approach offers a practical tool to enhance image quality in MRI of patients with metal hip implants.

## 1 Introduction

Due to an increasingly aging society, the prevalence of orthopedic implants, including total hip arthroplasty (THA), is increasing substantially [1]. In the United States, the annual number of primary total hip arthroplasty procedures rose from 305,082 in 2013 to 594,981 in 2022, and projections suggest it could reach approximately 2.17 million by 2040 [1]. MRI has become very useful in the diagnosis of post‐operative complications associated with joint replacement, such as aseptic loosening or periprosthetic joint infection [2, 3]. Nevertheless, there are challenges with MRI near metal implants: Metal has a higher magnetic susceptibility than soft tissues, which distorts the main magnetic field $B_{0}$ and leads to a shift in the resonance frequency in the surrounding tissue [4]. Since the resonance frequency of protons forms the basis for spatial encoding in MRI, metal artifacts such as signal pile‐up or loss due to local T2* reduction, as well as geometric distortion can occur [5].

The most commonly used advanced techniques for metal artifact reduction are based on fast or turbo‐spin‐echo (TSE) sequences employing view angle tilting (VAT), slice encoding for metal artifact correction (SEMAC), and multi‐acquisition variable resonance image combination (MAVRIC) [6, 7]. VAT can correct in‐slice artifacts but not through‐slice artifacts, while SEMAC and MAVRIC address both in‐ and through‐slice artifacts [7]. MAVRIC encodes a 3D volume several times with different carrier frequencies of the RF pulses to cover a broad off‐resonance‐induced frequency spectrum [8]. SEMAC, on the other hand, excites 2D slices with VAT and additional 3D‐slice encoding steps to resolve the distorted slice profiles [9]. Both approaches are able to resolve even far off‐resonant signals in so‐called spectral bins, which can then be re‐assigned with proper spatial locations in a post‐processing step. However, while the spectral bin recombination is very effective in correcting major image distortions, several residual artifacts may affect the resulting images. Most prominently, ripple artifacts and blurring are known side effects, making diagnosis in the affected regions challenging [10]. For SEMAC, it has been demonstrated that overlaps between slice profiles can attenuate ripple artifacts [11]. However, this method requires longer acquisition times and increases the specific absorption rate (SAR) of the sequence, making it impractical for clinical applications.

Ripple artifacts associated with SEMAC imaging display a specific frequency distribution, particularly prominent at higher spatial frequencies ($\sim 0.5-2.0\;cm^{-1}$) [12]. In the spatial domain, ripple artifacts manifest as a wave‐like pattern with an extent of a few centimeters. Utilizing the discrete 2D wavelet transformation, we can effectively analyze the characteristics of these artifacts. The 2D wavelet domain captures spatial frequencies in localized regions of the image at different scales and orientations [13, 14]. Consequently, wavelets can be employed to represent ripple artifacts in anatomical images.

In this work, a novel wavelet domain filter (WD‐Filter) was developed to reduce ripple artifacts in MRI of total hip arthroplasty without increasing acquisition time. This study involved 100 patients and evaluated the impact of the proposed filter on ripple artifact intensity and overall image quality in compressed‐sensing SEMAC images.

## 2 Materials and Methods

The retrospective study was approved by the local Ethics Committee (Cantonal ethics committee, Zurich, Switzerland) and written informed consent was obtained from all patients prior to the MRI examination.

### 2.1 Patient Selection

A total of 100 patients (51 females, mean age 64 years (±13 years) and 49 males, mean age 65 years (±13 years)) who underwent MRI of total hip arthroplasty for clinical indications were included in this study. Median time since total hip arthroplasty implantation was 2.0 years (interquartile range: 1.0–6.3 years). Implant materials varied and included ceramic, cobalt–chromium, and titanium femoral heads; primarily titanium femoral stems; and acetabular components composed of high nitrogen stainless steel with a titanium alloy, ceramic with a titanium alloy, titanium, or cobalt–chromium. Ripple artifacts were visually identified jointly by a musculoskeletal radiologist and MR scientist (S.S.G. and J.C.D. with 6 and 2 years of experience, respectively) on the CS STIR SEMAC images. Overall, ripple artifacts were observed in 66 out of 100 image datasets. These artifacts were evident in either one or multiple slices within an image dataset. If there were more than three slices with ripple artifacts per patient, we selected three slices for the correction. Within each selected slice, all identified ripple artifacts were addressed, with one slice potentially containing between one and four artifacts (Figure S2). For image analysis, each ripple artifact was considered as a separate unit for analysis.

### 2.2 Filter in Wavelet Domain

The filtering process for ripple artifact reduction consisted of three steps (Figure 1). The first step was identifying the ripple artifact in the in vivo image. A region of interest (ROI) was manually selected by an MR scientist (J.C.D., 2 years of experience) to specifically target the area associated with the ripple artifacts. Filtering was only used in the ROI to avoid filtering detailed structures within the whole field of view (FOV), as edges or transitions in MR images could also exhibit high absolute values for the detail coefficients (*cD*) [13, 15].

**FIGURE 1:**
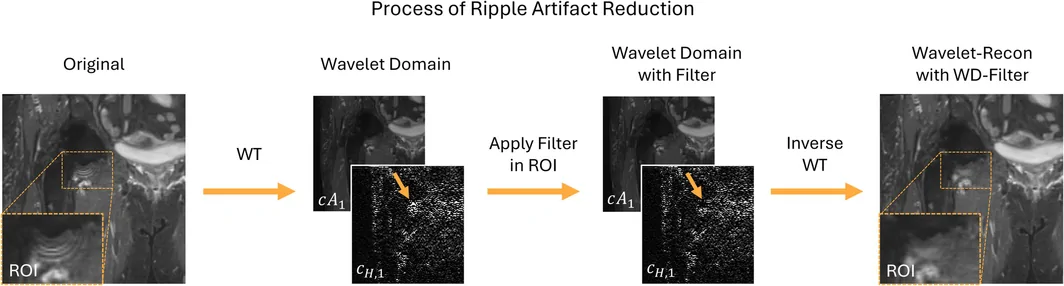
Process of ripple artifact reduction: The filtering process involves selecting the region of interest (ROI) containing the ripple artifact, as shown on a coronal compressed sensing STIR SEMAC MR image of total hip arthroplasty. A wavelet transform (WT) is then applied to the entire image, and a signal thresholding filter is employed within the ROI to remove high absolute detail coefficients corresponding to ripple artifacts. This example illustrates the horizontal detail coefficients of the first decomposition level ($c_{H,1}$). The yellow arrow points to coefficients associated with the ripple artifacts before and after the filtering process. The approximation plane (${\mathrm{cA}}_{1}$) does not involve any filtering. The resulting inverse wavelet transform yields an MR image with reduced ripple artifacts, that is, within the ROI no residual ripple artifact is seen in the final image. ROI, region of interest; SEMAC, slice encoding for metal artifact correction; STIR, short tau inversion recovery; WD‐Filter, wavelet domain filter.

Subsequently, image data were transformed from image domain to wavelet domain by a two‐dimensional (2D) discrete wavelet transformation (WT; Supporting Information) [14, 16]. This transformation divided the image into the approximation coefficients *cA* (representing low frequencies) and the detail coefficients *cD* (representing higher frequencies). The *cD* is further split into the horizontal ($c_{H}$), vertical ($c_{V}$), and diagonal ($c_{D}$) coefficients to identify changes or edges in their respective directions. Depending on the selected wavelet family, vanishing moments, and decomposition level, high absolute values for *cD* can be associated with the ripple artifacts [17].

The WD‐Filter used in this study involves the application of Fejér‐Korovkin wavelets (fk), with 6 or 12 vanishing moments (fk6 or fk12), and a decomposition level of 2, to capture all *cD* coefficients associated with the artifacts. Further details of the fk‐wavelet filter are described in the Supporting Information.

Following the WT, a binary mask based on signal thresholding was applied to each *cD*. The mask for each *cD* within the ROI had the following condition:

$$\mathrm{Mas}k_{\mathrm{ROI}}=\left\{\begin{array}{c} 0\;\text{if}\;\mathrm{abs}\left(c_{i,n}\right)>p_{i,n}*\max\left(\mathrm{abs}\left(c_{i,n}\right)\right)\\ 1\;\text{if}\;\mathrm{abs}\left(c_{i,n}\right)\leq p_{i,n}*\max\left(\mathrm{abs}\left(c_{i,n}\right)\right) \end{array}\right.$$

where c represents the detail coefficient for a specific direction i (horizontal H, vertical V, or diagonal D) and decomposition level n, while p denotes the threshold factor. The value $p_{i,n}$ was fixed for each $c_{i,n}$. The thresholds $p_{i,n}$ were determined by an musculoskeletal radiologist and two MR scientists (R.S.: 22 years, J.C.D.: 2 years, C.V.D.: 15 years of experience) based on visual inspection of ripple artifact reduction and retention of anatomical detail in ten artifact cases from five in vivo datasets. The optimal thresholds for ripple artifact reduction were $p_{H,1}=0.12$, $p_{V,1}=0.45$, $p_{D,1}=0.18$, $p_{H,2}=0.4$, $p_{V,2}=0.45$, $p_{D,2}=0.35$.

After applying the mask, the inverse wavelet transform was used to reconstruct the image. The WD‐Filter algorithm was implemented in MATLAB R2023b (MathWorks, Natick, MA, USA) using the Wavelet Toolbox.

### 2.3 Phantom and In Vivo MR Imaging

For the phantom measurements, a total hip arthroplasty consisting of a titanium stem and a cobalt‐chromium head (Medacta, Castel San Pietro, Switzerland) was embedded into a custom‐built brick structure positioned in a container filled with water. Phantom imaging was performed on a 1.5 T MRI scanner (MAGNETOM Avanto Fit; Siemens Healthineers, Forchheim, Germany) using a 32‐channel spine coil and an 18‐channel surface coil for signal reception. The imaging protocol (see Table 1) included a compressed sensing (CS)‐based coronal SEMAC sequence, with two opposite readout directions (H > F and F > H) to shift the ripple artifacts to various locations. Subsequently, the WD‐Filter was applied to the SEMAC images acquired in both directions, producing a total of four images for analysis: two images displaying ripple artifacts at distinct locations, and two corresponding images in which the ripple artifacts were effectively reduced upon filtering (Figure 2).

**TABLE 1:** Protocol for coronal STIR compressed‐sensing SEMAC sequence and coronal phantom SEMAC sequence.

	Patient MAGNETOM Sola	Patient MAGENTOM Avanto Fit	Phantom MAGENTOM Avanto Fit
Receive bandwidth (Hz/pixel)	539	501	546
SEMAC encoding steps	12	19	12
TR (ms)	5000	4220	4000
TE (ms)	37	36	38
TI (ms)	145	160	—
Slices	28		21
Slice thickness (mm)	3.5		3.5
FOV (mm^2^)	280 × 280		225 × 316
Pixel size (mm^2^)	0.9 × 0.9	1.1 × 1.1	0.9 × 0.9
Orientation	Coronal		Coronal
Phase encoding direction	R > > L		H > > F/F > > H
Flip angles (degrees)	120–180	120–140	120
Turbo factor	13	9	17
Acceleration mode	Compressed Sensing (CS)		CS
Undersampling factor	8		8
Acquisition time (min)	4:42	6:30	2:20

Abbreviations: FOV, field of view; SEMAC, slice encoding for metal artifact correction; TE, time to echo; TI, time of inversion; TR, time to repetition.

**FIGURE 2:**
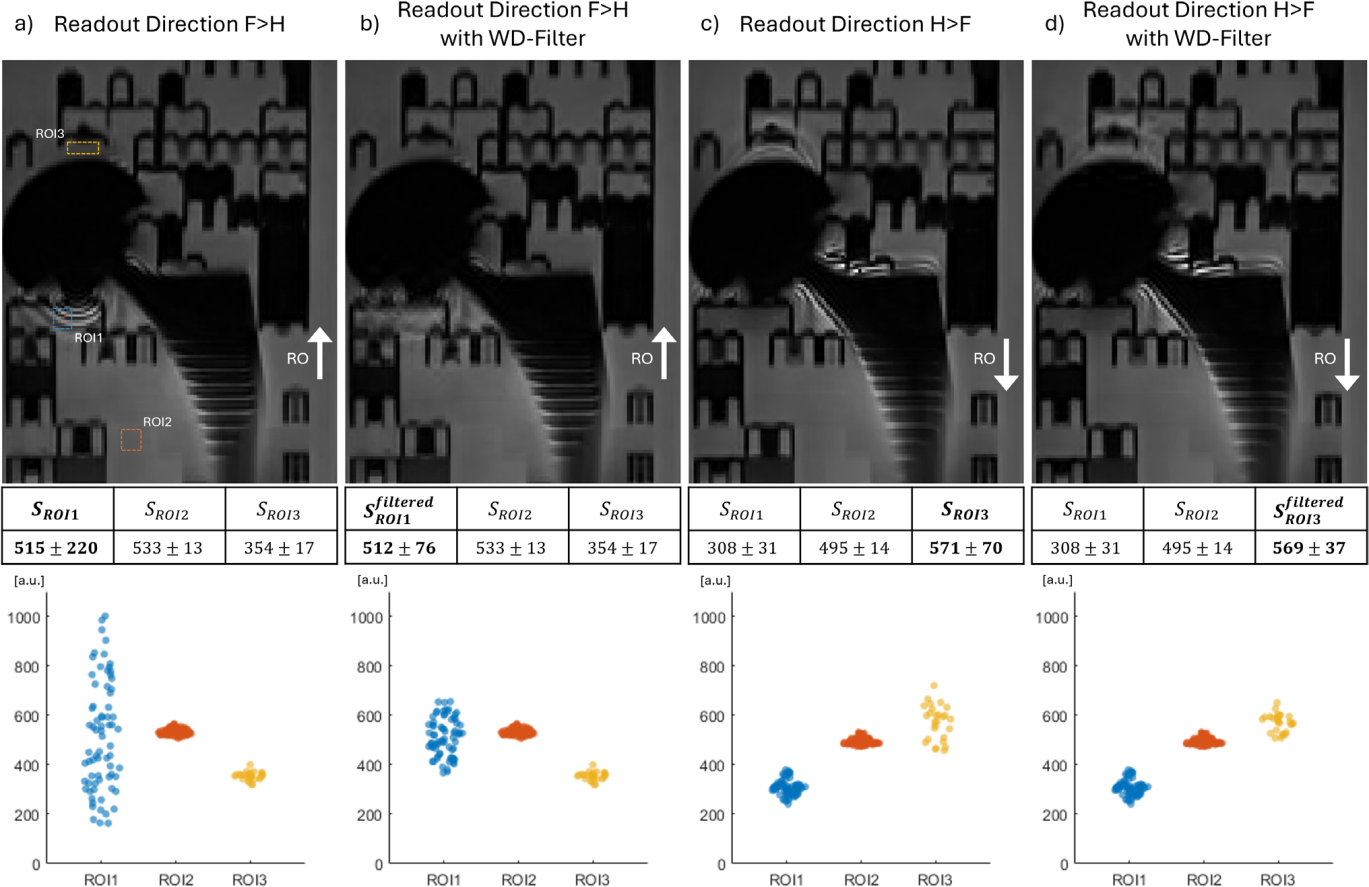
Phantom SEMAC images acquired with opposite readout directions. In (a), the image was obtained with a readout direction (RO) from feet to head (F > H), as indicated by the white arrow. This orientation causes ripple artifacts below the implant cup. Three representative regions of interest (ROIs) are highlighted in (a) and consistently applied across all images. (c) shows the image acquired in the reverse readout direction, from head to feet (H > F), resulting in the ripple artifact shifting above the implant cup. (b) and (d) present the corresponding images after applying the WD‐Filter, demonstrating a reduction of ripple artifacts. The table below each image shows the mean and standard deviation (mean±SD) of the signal intensity for each ROI in arbitrary units. The plots beneath each table illustrate voxel intensities for each ROI. Notably, the plots for the filtered images reveal a pronounced decrease in intensity variation within the affected regions, confirming effective suppression of ripple artifacts and improved image homogeneity. F > H, feet to head; H > F, head to feet; RO, readout; ROI, region of interest; SEMAC, slice encoding for metal artifact correction; WD‐Filter, wavelet domain filter.

For the retrospective study, only patients with primary total hip arthroplasties were selected. The images were acquired with two 1.5 T MRI scanners (MAGNETOM Sola; MAGNETOM Avanto Fit; Siemens Healthineers, Forchheim, Germany) at Balgrist University Hospital (Zurich, Switzerland) between December 2023 and June 2024 (Figure S1) using a standard clinical MR protocol for total hip arthroplasties (see Table 1). A 32‐channel spine coil combined with an 18‐channel surface coil was used for signal reception, and the imaging protocol included a compressed sensing (CS)‐based coronal short inversion time recovery (STIR) SEMAC sequence (Table 1) [18].

### 2.4 Image Analysis

Four phantom images (Figure 2) were analyzed by selecting three distinct ROIs in each image. ROI placement was performed by an MR scientist (J.C.D., 2 years of experience). Two of these ROIs included portions of a ripple artifact visible in at least one of the images. The third ROI was positioned in an area containing only water signal, free from both artifacts and brick, and served as a control region representing unaffected signal. For each ROI across all images, intensity values were individually plotted before and after the WD‐Filter application. The alternating signal of the ripple artifacts was also quantified by the mean signal intensity and standard deviation $\left(S_{\mathrm{ROI}}\right)$.

For the study, three musculoskeletal fellowship‐trained radiologists (S.S.G., G.W.K., A.A.M. with 5, 6, and 4 years of experience, respectively) independently evaluated the ripple artifacts, comparing the original image to the images processed with the WD‐Filter (Figure S1). To assess the severity of the ripple artifacts before and after filtering, a 4‐point Likert scale was used, labeled as Likert‐RA (Likert‐Ripple Artifact), to measure the intensity of ripple artifacts. The Likert‐RA scores were classified as 1 = none, 2 = mild, 3 = moderate, and 4 = severe. Figure 3 shows representative images with ripple artifacts for the Likert‐RA scores, where all readers scored the images identically. Additionally, a 4‐point Likert scale was employed to evaluate image quality (Likert‐IQ), with scores of 1 = poor, 2 = moderate, 3 = good, and 4 = excellent. Scores reflected the global subjective impression of diagnostic image quality, including sharpness, image noise, contrast, and artifacts.

**FIGURE 3:**
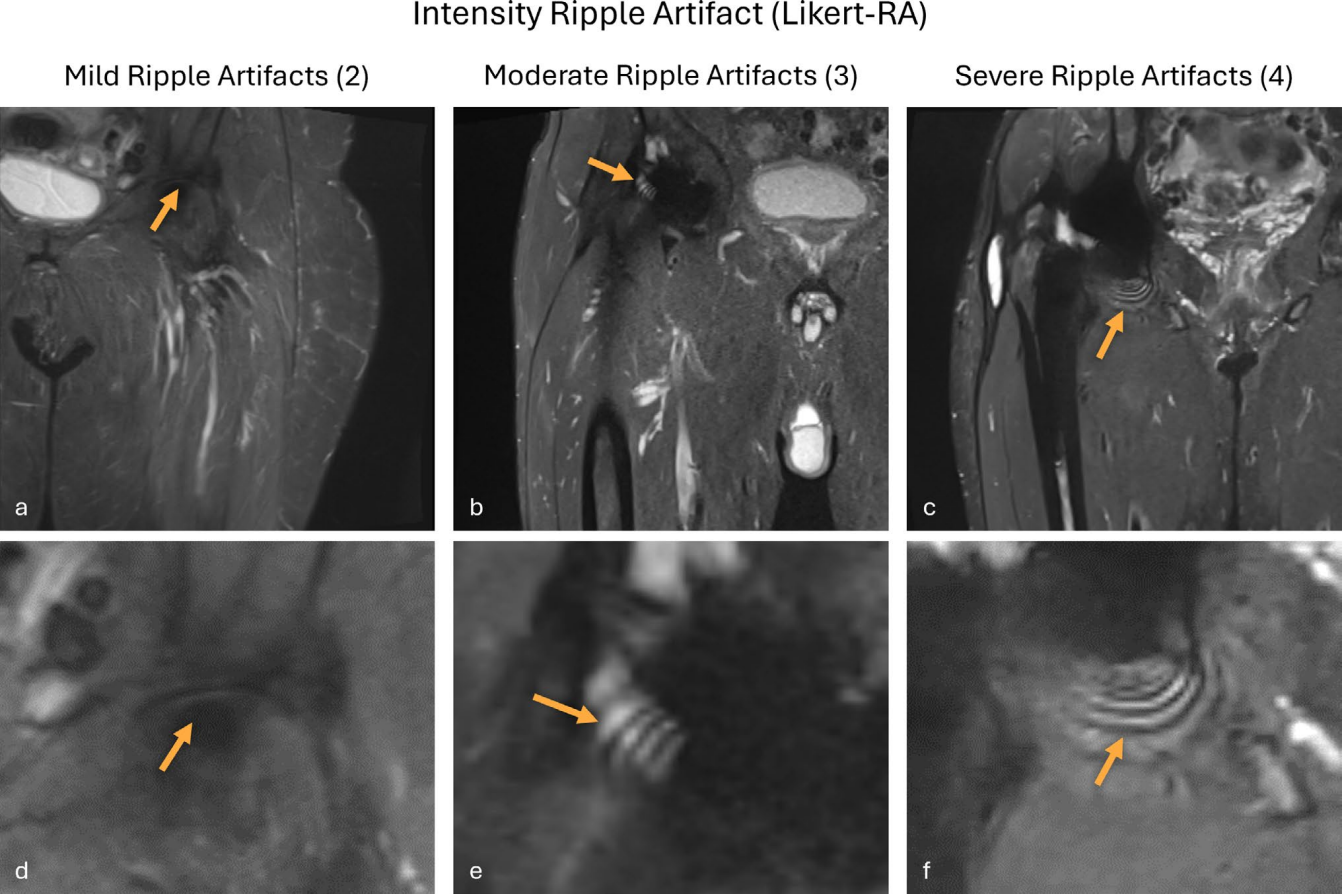
Examples of coronal STIR compressed sensing SEMAC images with rated Likert‐RA (ripple artifact) values in three different patients. The arrows are pointing to the ripple artifacts in all images. The first row (a–c) displays three different in vivo MR images with ripple artifacts, and the second row (d–f) shows the enlarged ROI of the same ripple artifacts. All radiologists rated these images with the same Likert values for the intensity of ripple artifacts: Image (a) was rated as 2 (mild), image (b) as 3 (moderate) and image (c) as 4 (severe). Note that for one patient (a, d) a total hip arthroplasty of the left hip was present, and the ripple artifact shown here was located at the anterior edge of the cup of the prosthesis. Two patients (b, e and c, f) had a total hip arthroplasty of the right hip. SEMAC, slice encoding for metal artifact correction; STIR, short tau inversion recovery.

The ripple artifacts demonstrate an alternating signal which can also be measured by the signal intensity standard deviation, SD. For quantitative artifact analysis, the SD was computed within a manually placed ROI around the artifact for the original and filtered images. Here, the relative decrease in $SD_{\mathrm{ROI}}$ was computed:

$$\Delta SD_{\mathrm{ROI}}=\frac{SD_{\mathrm{ROI}}-{\mathrm{SD}}_{\mathrm{ROI}}^{\text{filtered}}}{SD_{\mathrm{ROI}}}$$

where $SD_{\mathrm{ROI}}$ is the SD within the ROI in the original image, and ${\mathrm{SD}}_{\mathrm{ROI}}^{\text{filtered}}$ is the SD within the ROI in the image with the applied WD‐Filter. To ensure that the SD in areas not impacted by the WD‐Filter remains unchanged, an additional ROI in the musculoskeletal area (gluteus medius muscle) was designated for comparison (Figure S2). Within this ROI, the SD was also measured ($SD_{\mathrm{MS}}$) in the original image and the image with the applied WD‐Filter. Edge preservation was evaluated in one example by signal intensity profile comparison pre‐ and ‐post filter application in two regions close to the implant (Supporting Information; Figure S3).

### 2.5 Statistics

Variables are presented as mean ± standard deviation. Likert scores (RA and IQ) and SD before versus after filtering were compared using a Wilcoxon signed‐rank test and a two‐sided paired *t*‐test, respectively. A *p* value of less than 0.05 was considered statistically significant [19]. For the Wilcoxon signed‐rank test, effect size r was calculated and interpreted as small (r=0.10−0.29), medium (r=0.30−0.49), or large (r≥0.5) [20, 21]. The inter‐reader agreement for the Likert‐RA and Likert‐IQ scores was assessed using kappa statistics (Fleiss κ) with 95% confidence interval. The level of agreement with a κ value of 0.00 indicated “poor”, 0.00–0.20 “slight”, 0.21–0.40 “fair”, 0.41–0.60 “moderate”, 0.61–0.80 “substantial”, and values from 0.81 to 1.00 “almost perfect” agreement [22]. All statistical analyses were performed using SPSS Statistics (version 31, IBM).

## 3 Results

### 3.1 Phantom

The WD‐Filter effectively reduced artifacts in the phantom images (Figure 2). After filtering, intensity values within artifact‐affected ROIs became more consistent and aligned closely with those in the water signal control regions in ROI2 $\left(S_{\mathrm{ROI}2}=\left(495-533\pm 14-13\right)\right)$ ROI1 exhibited a wide range of values due to ripple artifacts ($S_{\mathrm{ROI}1}=\left(515\pm 220\right),$ Figure 2a), however upon filtering (Figure 2b) these values became more uniform and similar to the reference ($S_{\mathrm{ROI}1}^{\text{filtered}}=\left(512\pm 76\right)$). Although ROI3 did not display visible artifacts in either image ($S_{\mathrm{ROI}3}=\left(354\pm 17\right)$ and $S_{\mathrm{ROI}3}=\left(354\pm 17\right)$), a decrease in signal relative to ROI2 was observed, likely because of dephasing effects near the implant. Comparing images with opposite readout directions (Figure 2c,d), the ripple artifact in Figure 2c was less intense than in Figure 2a but still present, as indicated by the broader value distribution in ROI3 ($S_{\mathrm{ROI}3}=\left(571\pm 70\right)$) After applying the WD‐Filter (Figure 2d), ROI3 values became more compact and approached the reference values ($S_{\mathrm{ROI}3}=\left(569\pm 37\right)$).

### 3.2 Prevalence of Ripple Artifact

For the study, ripple artifacts were detected in 66 out of 100 in vivo image datasets. Following the predefined selection and analysis strategy, between 1 and 10 ripple artifacts per patient were included. Overall, 165 slices were analyzed, resulting in a total of 250 ripple artifacts. The Likert‐RA and Likert‐IQ evaluations for all readers are summarized in Table 2.

**TABLE 2:** Evaluation of 250 ripple artifacts by three readers.

(a) Likert‐RA		Reader 1		Reader 2		Reader 3	
		Without WD‐filter	With WD‐filter	Without WD‐filter	With WD‐filter	Without WD‐filter	With WD‐filter
Number of ROI's with Likert‐RA score	1 = None	**0**	174	**0**	164	**0**	166
	2 = Mild	34	76	32	86	31	84
	3 = Moderate	92	**0**	94	**0**	100	**0**
	4 = Severe	124	**0**	124	**0**	119	**0**
Mean		3.4	1.3	3.4	1.3	3.4	1.3
Standard deviation		0.7	0.5	0.7	0.5	0.6	0.5
*p* value		< 0.001		< 0.001		< 0.001	
Effect size r		0.89		0.89		0.89	

(b) Likert‐IQ		Reader 1		Reader 2		Reader 3	
		Without WD‐filter	With WD‐filter	Without WD‐filter	With WD‐filter	Without WD‐filter	With WD‐filter
Number of ROI's with Likert‐RA score	1 = Poor	157	**0**	156	**0**	155	**0**
	2 = Moderate	85	15	89	15	90	16
	3 = Good	8	131	5	125	5	124
	4 = Excellent	**0**	104	**0**	110	**0**	110
Mean		1.4	3.4	1.4	3.4	1.4	3.4
Standard deviation		0.6	0.6	0.5	0.6	0.5	0.6
*p* value		< 0.001		< 0.001		< 0.001	
Effect size *r*		0.89		0.89		0.89	

*Note:* For better visualization, categories without artifacts have been labeled with a bold‐face zero.

Abbreviations: Likert‐IQ, Likert‐rating for image quality; Likert‐RA, Likert‐rating for ripple artifacts; WD‐Filter, wavelet domain filter.

A total of 57±2 (mean ± SD) ROIs in the original images, initially rated with a Likert‐RA score of 4 (severe), improved to a Likert‐RA score of 1 (none) after applying the WD‐Filter. Additionally, $\left(46\pm 2\right)$ ROIs upon filtering, were rated with an excellent Likert‐IQ (score 4) while original images initially were classified with a poor Likert‐IQ score of 1. An example of these improvements in both Likert scales is shown in Figure 4, where all readers evaluated the images identically.

**FIGURE 4:**
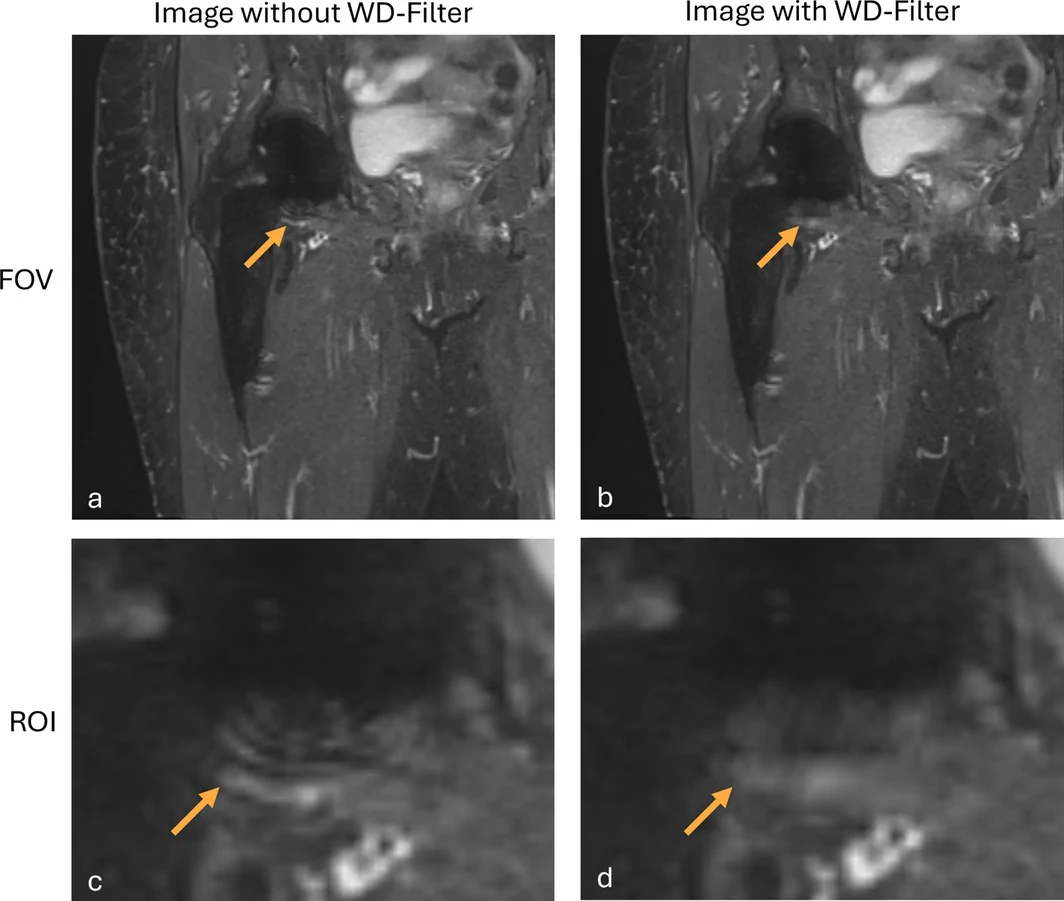
Ripple artifact reduction on MRI of the right hip in a patient with total hip arthroplasty. The arrows are pointing to the area affected by the ripple artifacts on uncorrected images (a, c), corrected images (b, d), and images with the enlarged ROI (c, d). Image (a) was rated with a Likert‐RA value of 4 (severe) and Likert‐IQ value of 1 (poor) by all readers. Image (b) was rated with a Likert‐RA value of 1 (none) and Likert‐IQ value of 4 (excellent). FOV, entire field‐of‐view; Likert‐IQ, Likert‐rating for image quality; Likert‐RA, Likert‐rating for ripple artifacts; ROI, region of interest; WD‐Filter, wavelet domain filter.

Furthermore, multiple ROIs in the WD‐Filter were used to successfully reduce more than one ripple artifact in each slice. Figure 5 illustrates a significant improvement in the Likert‐RA and Likert‐IQ values for two ripple artifacts. In addition, improved depiction of a cyst located superior to the acetabular component is observed in the filtered images, whereas artifact‐related distortion in the original images limits its depiction.

**FIGURE 5:**
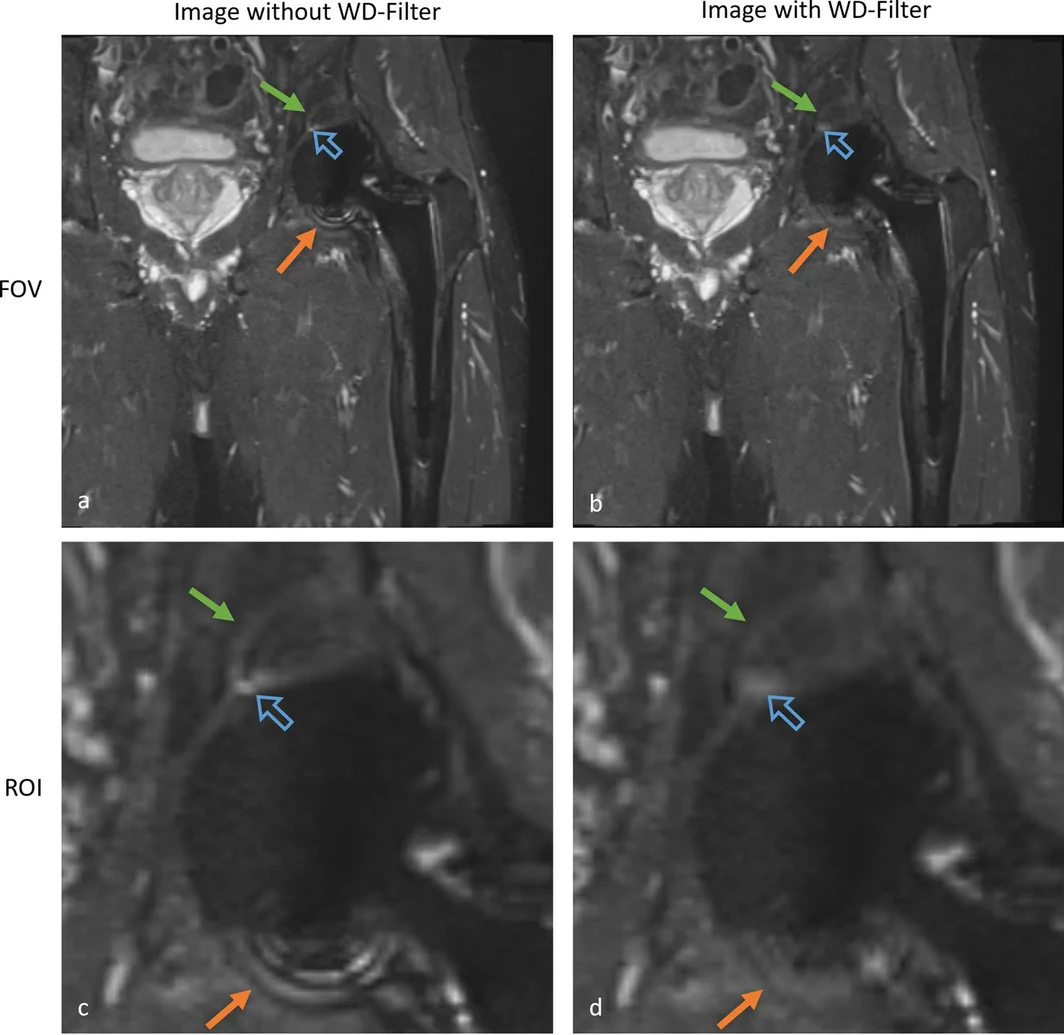
Ripple artifact reduction on MRI of the left hip in a patient with total hip arthroplasty. The solid arrows are pointing to two different ripple artifacts on uncorrected images (a, c), corrected images (b, d), and images with the enlarged ROI (c, d). The first ripple artifact (green arrow) was rated with a Likert‐RA value of 2.7 (reader 1: 2; reader 2, 3: 3) for the original image and a value of 1 for the image with WD‐Filter for all readers. Regarding Likert‐IQ, the uncorrected image was rated with a value of 2, and the corrected image with a value of 4. The second ripple artifact (orange arrow) was rated with a Likert‐RA value of 4 for the original image and a value of 2 for the corrected image. Regarding Likert‐IQ, the uncorrected image was rated with a Likert value of 1, and the corrected image with a value of 3. The outlined blue arrows highlight the improved visualization of a cyst located superior to the acetabular component in the corrected images (b, d), compared with the uncorrected images (a, c), where artifacts impair clear delineation. Likert‐RA, Likert‐rating for ripple artifacts (1 = none, 4 = severe); Likert‐IQ, Likert‐rating for image quality (1 = poor, 4 = excellent); FOV, entire field‐of‐view; ROI, region of interest; WD‐Filter, wavelet domain filter.

In some instances, residual artifacts were still visible after the WD‐Filter was applied (Figure 6). Nevertheless, there are no cases where a worse Likert value or a lack of improvement in image quality was seen after applying the WD‐Filter. For each ripple artifact in the original image, an enhancement of at least one point on the Likert scale value was achieved after the wavelet domain filtering.

**FIGURE 6:**
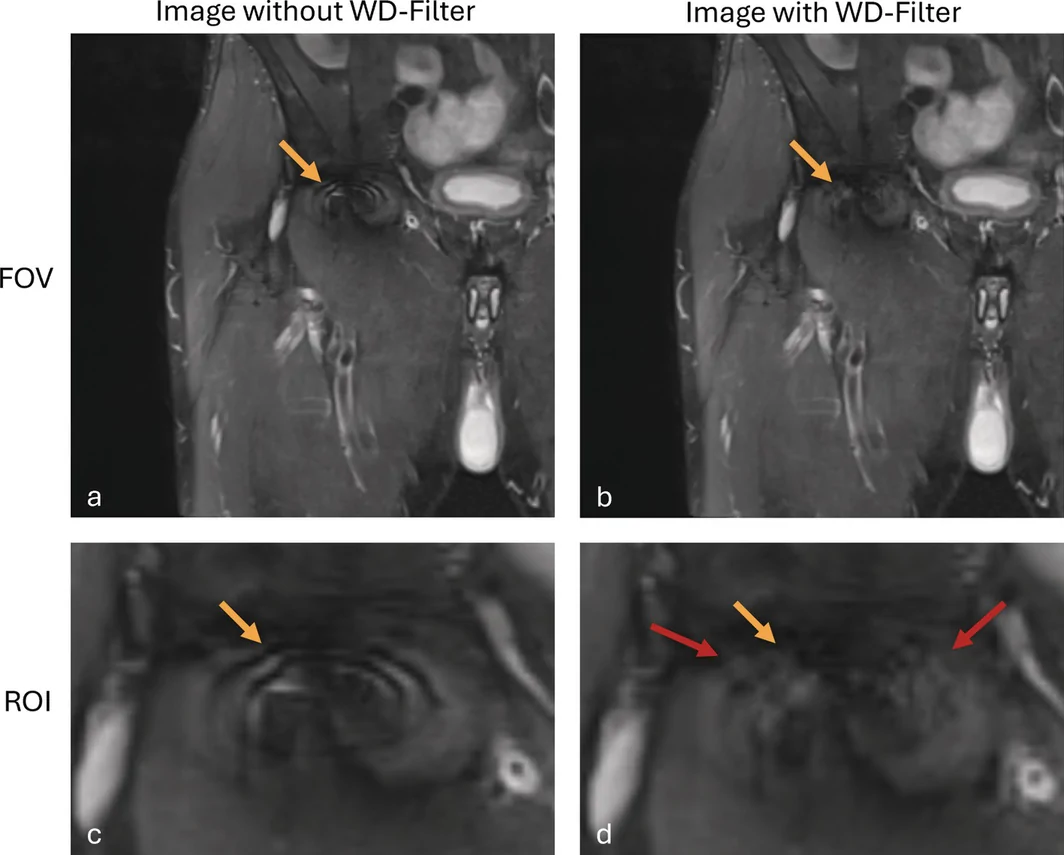
Ripple artifact reduction. The orange arrows are pointing to the ripple artifacts and the red arrows to the residual artifacts in the corrected image on MRI of the right hip in a patient with total hip arthroplasty; the ripple artifact shown here was located at the anterior edge of the cup of the prosthesis. The uncorrected images (a and c) are on the left side and the corrected images (b and d) on the right side. The original image (a) was rated with a Likert‐RA value of 4 and Likert‐IQ value of 1 regarding image quality, and the corrected image (b) was rated with a Likert‐RA value of 2 and Likert‐IQ value of 2 regarding image quality. Likert‐RA, Likert‐rating for ripple artifacts (1 = none, 4 = severe); Likert‐IQ, Likert‐rating for image quality (1 = poor, 4 = excellent); FOV, entire field‐of‐view; ROI, region of interest; WD‐Filter, wavelet domain filter.

### 3.3 Rating: Intensity of Ripple Artifacts

The three readers' ratings for the intensity of ripple artifacts are summarized in Table 2a. In the original image, (122±2) ripple artifacts were rated as “severe” (Likert‐RA = 4), (95±3) cases were rated as “moderate” (Likert‐RA = 3), and (32±1) cases as “mild” (Likert‐RA = 2). Upon wavelet domain filtering, the artifact was eliminated in (168±4) (Likert‐RA = 1). An additional (82±4) cases were rated as having “mild” residual ripple artifacts (Likert‐RA = 2). Finally, no ripple artifact was rated as “moderate” or “severe” after application of the WD‐Filter (Table 2a).

The mean Likert‐RA score for the original MR image was $\left(3.4\pm 0.7\right)$, while the filtered MR images had a mean Likert‐RA score of $\left(1.3\pm 0.5\right)$ across all readers. The Likert‐RA value significantly improved for all readers, with a large effect size (r=0.89). The inter‐reader agreement for the Likert‐RA scale was “almost perfect” at $\left(0.86\pm 0.03\right)$ for the original images and $\left(0.90\pm 0.04\right)$ for the images with the WD‐Filter.

### 3.4 Rating: Image Quality

Image quality was rated as “poor” (Likert‐IQ = 1) in (156±1) ROIs across readers. Rating of “moderate” (Likert‐IQ = 2) was assigned to (88±3) ROIs, while “good” quality (Likert‐IQ = 3) was noted in (6±2) ROIs.

After applying the WD‐Filter, (108±3) cases were rated as “excellent” (Likert‐IQ = 4). An additional (127±4) ROIs, were rated as “good” (Likert‐IQ = 3), and (15±1) as “moderate” (Likert‐IQ = 2) (Table 2b).

The mean Likert‐IQ score improved from $\left(1.4\pm 0.6\right)$ to $\left(3.4\pm 0.6\right)$ upon filtering. The Likert‐IQ score significantly improved for all readers, with a large effect size (r=0.89). Regarding the Likert‐IQ scale, the inter‐reader agreement was “almost perfect” at $\left(0.95\pm 0.03\right)$ for the original images and $\left(0.96\pm 0.03\right)$ for the images with the WD‐Filter.

### 3.5 Quantitative Artifact Assessment

Reader 1 measured a reduction of $\left(19\%\pm 9\%\right)$ in $\Delta SD_{\mathrm{ROI}}$ after the WD‐Filter, while reader 2 and reader 3 reported reduction of $\left(19\%\pm 11\%\right)$ and $\left(19\%\pm 10\%\right)$, respectively. All readers observed a significant reduction in the value of ${\mathrm{SD}}_{\mathrm{ROI}}^{\text{filtered}}$ for the corrected image compared to the original image, decreasing from $\left(73\pm 50\right)$ to $\left(58\pm 41\right)$. In contrast, ROIs placed in the skeletal muscle before and after applying the WD‐Filter showed no significant difference (p≥0.09) in $SD_{\mathrm{MD}}$ ($\left(10.1\pm 5.3\right)$ without WD‐Filter vs. $\left(10.1\pm 5.2\right)$ with WD‐Filter across readers).

## 4 Discussion

Hip arthroplasty and other metallic implants may cause severe metal artifacts in MRI scans. Several studies have highlighted the performance of SEMAC over standard MR sequences for metal artifact reduction [23, 24, 25, 26]. However, SEMAC can lead to the occurrence of detrimental ripple artifacts in areas near the implant due to the bin combination of SEMAC encodings [9, 11].

To minimize these artifacts, we developed a filter in the wavelet domain (WD). Phantom measurements demonstrated that applying the WD‐Filter effectively reduced ripple artifacts and minimized fluctuations in signal intensity. The WD‐Filter not only diminished the artifacts but also restored the appearance of the water signal, making it comparable to images obtained with the opposite readout direction. To assess its clinical impact, we tested the WD‐Filter in patients with total hip arthroplasty.

The current study addressed 250 image regions with ripple artifacts applying the WD‐Filter, which successfully reduced the severity of these artifacts. With the WD‐Filter, most MR images showed only “mild” or “no ripple artifact”. The average Likert‐RA score improved significantly and there was a significant reduction in the SD within the ROI containing the artifact, confirming the WD‐Filter's effectiveness. After reducing the ripple artifact, it is crucial to ensure that the image retains its diagnostic value. In this context, the WD‐Filter has shown its potential by improving the image quality, as reflected by the significant improvement in Likert‐IQ scores.

There are other approaches to reducing ripple artifacts in MR imaging. In order to achieve a homogeneous image combination, one approach is to increase the width of the slice profile for SEMAC to achieve a slice overlap [11, 27]. This can be achieved by either increasing the bandwidth of RF while maintaining the slice selection gradient or by keeping the RF bandwidth constant and reducing the gradient. However, both approaches have drawbacks, including increased specific absorption rate (SAR) or the need for more SEMAC encoding steps, increasing the acquisition time. Another potential approach is to incorporate a $B_{0}$ field map during image postprocessing, which has been shown to reduce ripple artifacts and signal displacement [28]. However, it is important to note that acquiring an additional field map prolongs acquisition time, and this method is unable to fully correct for signal fluctuation caused by slice selection profile variations, potentially resulting in residual artifacts.

The developed WD‐Filter is applied after the data acquisition and in the wavelet domain. Wavelet domain filtering is commonly applied in MRI for denoising or compressed sensing [29, 30]. The wavelet transformation (WT) divides anatomical images into approximation coefficients (low frequencies) and detail coefficients (high frequencies). This transformation enables the visualization of characteristics such as noise and allows for differentiation from the image. Depending on the wavelet family used for the WT, different image features become more distinguishable from the main structure of the image [17]. In our approach, we utilized the wavelet domain to distinguish ripple artifacts from the main structures of the image. By selectively filtering the detail coefficients in a region of interest using a threshold based on the maximum absolute value of the coefficient, it is possible to set the detail coefficients corresponding to the ripple artifact to zero while maintaining other coefficients to preserve image detail. Using the inverse WT with the unmodified approximation coefficients and the filtered detail coefficients produces an image with reduced ripple artifacts, allowing the anatomical structures underneath the artifacts to become visible.

### 4.1 Limitations

The WD‐Filter has only been tested using two different 1.5 T MRI scanners from the same vendor in a single‐center setting, as well as two different CS‐SEMAC protocols (12 and 19 SEMAC encoding steps) in patients with total hip arthroplasty. Reader blinding was not fully feasible, as localization of artifact regions required reference to unfiltered images, given that the artifacts were often absent after filtering. In 6% of cases, the corrected images only achieved a “moderate” image quality due to residual artifacts, indicating that the WD‐Filter may not fully address certain shapes or orientations of ripple artifacts. Adjusting the WD‐Filter's threshold factors ($p_{i,n}$) or considering a different wavelet could mitigate this issue. One drawback of our approach is the manual ROI placement during the filtering process. Consequently, all ripple artifacts in each slice must be manually delineated to obtain a filtered image, which may limit clinical workflow efficiency. Despite these limitations, our WD‐Filter may be the basis for potential automated ripple reduction approaches in the future using deep learning algorithms [31, 32]. While training a deep learning model to reduce ripple artifacts may be challenging due to the lack of ground truth images, the proposed WD‐Filter provides images without ripple artifacts or with reduced ripple artifacts, which may be used for artifact reduction model training. Alternatively, a DL model could be trained for ripple artifact segmentation to automate the ROI placement.

So far, the WD‐Filter has only been evaluated in patients with total hip arthroplasty imaged at 1.5 T. However, ripple artifacts have also been reported at 0.5 and 3 T field strengths as well as in the presence of different implant types and anatomical regions [3, 6, 33, 34]. As these artifacts arise from strong B0 field inhomogeneities in the in‐plane and through‐plane directions, their appearance and intensity may vary depending on field strength, implant shape, orientation, and composition [11]. Since the WD‐Filter targets extreme absolute detail coefficients in the wavelet domain that are directly associated with the artifact, our proposed approach is expected to remain applicable under varying conditions, although parameter tuning may be required.

To better understand the WD‐Filter's capabilities, successive studies should assess the effectiveness of the WD‐Filter across different field strengths and in patients with other metal implants such as knee arthroplasty [3, 24] or to other multispectral imaging techniques like MAVRIC [8].

## 5 Conclusion

The developed WD‐Filter successfully reduces ripple artifacts, enhancing the visibility of the tissue near the implant and improving image quality in MR imaging for patients with metal hip implants. While metal artifact reduction sequences generally have longer acquisition times than standard MR sequences, the application of the WD‐Filter during post‐processing allows for its inclusion in daily clinical practice without changing protocols or acquisition times.

## Conflicts of Interest

This research project is partly funded by Balgrist University Hospital, and by Siemens Healthineers International AG, Switzerland: Jeanette C. Deck is employed part‐time by Balgrist University Hospital and part‐time as a research fellow at Siemens Healthineers International AG. Constantin von Deuster is an on‐site‐scientist of Siemens Healthineers International AG at the Swiss Center for Musculoskeletal Imaging (SCMI) at Balgrist Campus. Balgrist Campus AG and Balgrist University Hospital, each have a general research agreement with Siemens Healthineers International AG. Balgrist University Hospital also has an academic research collaboration with Bayer.

## Supporting information


**Figure S1:** Flowchart illustrating patient inclusion and exclusion criteria. Number of patient N and number of ROIs with a ripple artifact $N_{\mathrm{ROI}}$. Ripple artifacts were corrected in a maximum of three slices for each patient. In each selected slice, all visible ripple artifacts were addressed. Each individual artifact was counted as a unit, leading to a total of 250 ripple artifacts analyzed in this study.
**Figure S2:** Ripple artifact selection on MRI of the right hip in a patient with total hip arthroplasty. Ripple artifacts are identified within a single MRI slice. It is possible for a single slice to include multiple ripple artifacts, as depicted in this figure, where three separate ripple artifacts were present. Each individual ripple artifact was considered a separate unit in the analysis, denoted as ROI1, ROI2, and ROI3. Initially, each ROI was positioned within the original image (a) containing a single ripple artifact. Subsequently, the ROI was copied and placed in the same location within the image that has undergone wavelet domain filtering (b). All ROIs were independently assessed by all readers, evaluating the intensity of the ripple artifact (Likert‐RA), image quality (Likert‐IQ), and the standard deviation of the signal intensity ($SD_{\mathrm{ROI}}$). The blue circle marks the reference ROI in the gluteus medius muscle used for evaluating the standard deviation of the signal intensity in muscle ($SD_{\mathrm{MS}}$) for one slice. ROI, region of interest.
**Figure S3:** Structural preservation of anatomical details in coronal STIR SEMEAC images of a patient with total hip arthroplasty. (a) Image without WD‐Filter and (b) image with WD‐Filter. Line profiles across the interface between bone and the acetabular component near two ripple artifacts (Position 1 and 2) compare the radial signal intensities without (red lines in a) and with filtering (green lines in b). (c) Signal profiles corresponding to position 1 and (d) signal profiles corresponding to position 2. Similar profiles were seen with and without filtering, indicating a well‐defined edge preservation. Pos, position; WD‐Filter, wavelet domain filter.
