# Supplementary material for: MRI of Total Hip Arthroplasty: Successful Ripple Artifact Reduction Using Wavelet Domain Filtering
Source: J Magn Reson Imaging. 2026 Jul 11;64(5):1326–36. doi: 10.1002/jmri.70448 (PMC13667339; doi:10.1002/jmri.70448)
Supplement: Supplementary file 1 [file JMRI-64-1326-s001.docx]

**Supplemental Material**

**Ripple Artifact Representation in the Wavelet Transformation:**

Ripple artifacts associated with SEMAC imaging display a specific frequency distribution, particularly prominent at higher spatial frequencies ($\sim0.5-2 cm^{-1}$) (12). Addressing these artifacts involves filtering in the Fourier domain, requiring a broad mask for higher frequencies. However, this approach also filters out image information, resulting in a significant degradation of image quality. In the spatial domain, ripple artifacts manifest as a wave-like pattern with an extent of a few centimeters. Utilizing the discrete 2D wavelet transformation, we can effectively analyze the characteristics of these artifacts.

In case of 2D images, the WT provides spatial frequency distribution in the corresponding wavelet domain (13). This is accomplished through the analysis of localized regions within the image at various scales and orientations. Consequently, wavelets can be employed to represent ripple artifacts in anatomical images.

The WT involves the application of a filter (wavelet filter banks) to the image. This filter includes scaling and wavelet functions, for the decomposition of the image into different frequency bands. The transformation results in the division of the image into an approximation plane (low frequencies) and three detail bands (high frequencies), involving information in horizontal, vertical and diagonal orientations. The approximation coefficients (*cA*) summarize the main structures of the image, while the detail coefficients (*cD*) capture the finer details. The horizontal ($c_{H}$), vertical ($c_{V}$) and diagonal ($c_{D}$) coefficients identify changes or edges in their respective directions. Notably, high absolute values in the detail coefficients indicate pronounced edges, transitions or alterations, whereas low absolute values signify minimal or no changes in the image, indicating homogeneous areas.

Employing a multilevel 2D-WT involves repeating the process with the approximation plane, leading again to a decomposition of approximation and detail coefficients. Each level reflects a lower resolution of the image and reveals larger structures within the image. The choice of the wavelet family influences the details visible in the image transform (17). For our specific purpose, we found that Fejér-Korovkin Wavelets (fk) yield high absolute values for various shapes of ripple artifacts. Additionally, the number of vanishing moments for the wavelet must be carefully determined. More vanishing moments facilitate the detection of high-frequency components such as edges and details, whereas fewer vanishing moments offer improved time localization and are advantageous for rapid changes.

Our analysis of ten ripple artifacts within five in vivo MRI datasets revealed that wavelets with 6 or 12 vanishing moments (fk6, fk14) most effectively captured high absolute values for the detail coefficients associated with the ripple artifacts. Furthermore, we observed that high absolute coefficient values correlated with artifacts that were associated with two decomposition levels. This insight was important for constructing an effective filter in the wavelet domain to target the coefficients linked to the ripple artifact.

**Supplementary figures:**

**Figure S1:** Flowchart illustrating patient inclusion and exclusion criteria. Number of patient N and number of ROIs with a ripple artifact $N_{ROI}$. Ripple artifacts were corrected in a maximum of three slices for each patient. In each selected slice, all visible ripple artifacts were addressed. Each individual artifact was counted as a unit, leading to a total of 250 ripple artifacts analyzed in this study.

**Figure S2:** Ripple artifact selection on MRI of the right hip in a patient with total hip arthroplasty. Ripple artifacts are identified within a single MRI slice. It is possible for a single slice to include multiple ripple artifacts, as depicted in this figure, where three separate ripple artifacts were present. Each individual ripple artifact was considered a separate unit in the analysis, denoted as ROI1, ROI2, and ROI3. Initially, each ROI was positioned within the original image (**a**) containing a single ripple artifact. Subsequently, the ROI was copied and placed in the same location within the image that has undergone wavelet domain filtering (**b**). All ROIs were independently assessed by all readers, evaluating the intensity of the ripple artifact (Likert-RA), image quality (Likert-IQ), and the standard deviation of the signal intensity ($SD_{ROI}$). The blue circle marks the reference ROI in the gluteus medius muscle used for evaluating the standard deviation of the signal intensity in muscle ($SD_{MS})$ for one slice. ROI, region of interest.

**Figure S3**: Structural preservation of anatomical details in coronal STIR SEMEAC images of a patient with total hip arthroplasty. **a)** Image without WD-Filter and **b)** image with WD-Filter. Line profiles across the interface between bone and the acetabular component near two ripple artifacts (Position 1 and 2) compare the radial signal intensities without (red lines in a) and with filtering (green lines in b). **c)** Signal profiles corresponding to position 1 and **d)** signal profiles corresponding to position 2. Similar profiles were seen with and without filtering, indicating a well-defined edge preservation. Pos, position; WD-Filter, wavelet domain filter.
